# Chromatin and Transcriptional Tango on the Immune Dance Floor

**DOI:** 10.3389/fimmu.2014.00631

**Published:** 2014-12-15

**Authors:** Ananda L. Roy, Robert G. Roeder

**Affiliations:** ^1^Programs in Immunology and Genetics, Department of Developmental, Molecular and Chemical Biology, Tufts University School of Medicine, Boston, MA, USA; ^2^Laboratory of Biochemistry and Molecular Biology, The Rockefeller University, New York, NY, USA

**Keywords:** immune response, transcription, promoter, enhancer, chromatin

The process of generating differentiated cell types performing specific effector functions from their respective undifferentiated precursors is dictated by extracellular signals, which alter the host cell’s capacity to perform cellular functions. One major mechanism for bringing about such changes is at the level of transcription. Thus, the transcription-related induction of previously silent genes and suppression of active genes in response to extracellular signals can result in the acquisition of new functions by the cells. The general transcriptional machinery, which comprised of RNA Polymerase II and associated initiation factors, assemble into preinitiation complexes at the core promoters of eukaryotic protein coding genes in response to the signal-dependent activation of corresponding regulatory factors that bind to promoter and enhancer elements (1). The rate of formation and/or stability of these complexes, which can be modulated both by enhancer–promoter interactions and by chromatin structural modifications, dictate the transcriptional regulation of the corresponding gene. Such coordinated temporal and spatial regulation of gene expression in response to specific signals determines lineage differentiation, cellular proliferation, and development (2).

Every event in the life cycle of a lymphocyte is modulated by the signals they receive. For instance, expression of the B cell antigen receptor (BCR) on the surface of B cells is a hallmark of various stages of B cell development, with signaling through the BCR being important during both early/antigen-independent (tonic) and late/antigen-dependent phases of development (3). However, how BCR signaling connects to chromatin changes and downstream transcriptional pathways at each step of development remains poorly understood. Similar questions also remain in other cells of the immune system. In particular, how enhancers communicate with promoters in a stage-specific fashion and in the context of chromatin also remain unclear (2). Chromatin modifiers are generally present and active in most cell types (4, 5). How then could there be gene-specific differences in chromatin architecture dependent on a particular stage of development?

The B (and T) lymphocytes also perform a unique developmental program because they have an unparalleled genetic makeup – the genetic loci that encode their cell surface receptors are in an “unrearranged” or “germline” configuration during the early stages of development. Thus, while expressing stage-specific genes and transcription factors during each developmental stage, lymphocytes also need to undergo rearrangement of their cognate receptor loci in a strictly ordered fashion to generate a pool of receptor proteins that, individually, are capable of recognizing specific antigens that are encountered at a much later step (6). Hence, there must be a strict negotiation between the recombination machinery and the transcriptional machinery at every developmental step. Importantly, along the way, those B cells that express receptors capable of recognizing self-antigens must be eliminated to avoid autoimmune responses and only those cells capable of recognizing foreign-antigens are preserved for migration to peripheral organs where they eventually encounter pathogens. How are these processes coordinately regulated in a stage-specific fashion and what role does chromatin play? Are the rules of engagement different in innate versus adaptive immune responses? The following 15 articles address some of these questions and provide important insights regarding our current understanding of signal-induced chromatin and transcriptional regulation of the immune system.

## Regulation of V(D)J Recombination – Role of Transcription and Chromatin

Germline configurations of antigen receptor loci in B and T lymphocytes have hundreds of variable (V) region gene-segments, which have the potential to combine with a select few diversity (D) and joining (J) gene-segments to create recombined genes encoding numerous receptors that can recognize a vast repertoire of antigens (6, 7). Given the importance and timing of these events, it is no wonder that the process of “V(D)J recombination” is exquisitely regulated at multiples levels. Two exciting articles, one by Chaumeli and Skok (8) and the other by Choi and Feeney (9), review our current understanding of how transcription factors, chromatin architecture, and the three-dimensional architecture of the nucleus and the topology of genomic DNA regulate this process. An interesting article by Basu and colleagues describes how ubiquitination events regulate the RAG and activation-induced cytidine deaminase (AID) enzymes that are important for recombination (10). Moreover, this article also discusses how these post-translational events also regulate DNA damage at undesirable loci and during cell cycle phases (10).

## Transcription Factors in Hematopoietic Development

Recombination and transcription are coupled during hematopoietic development (11–13). The next set of articles deal with factors involved in this coordination. Atchison and colleagues describes the role of an important but ubiquitously expressed transcription factor YY1 in this highly tissue-specific function (14). Clark and colleagues review the function of interleukin-7 receptor (IL7R) and transcription factor STAT5 in balancing proliferation and recombination of the immunoglobulin light chain (Igκ) gene (15). Bergman and colleagues present primary studies on the role of another essential transcription factor Pax5 in regulating the Igκ gene (16). The sequential involvement of transcription factors and chromatin regulators remains an open question, and Choukrallah and Matthias review our current understanding of these factors in B cell development (17). Webb and colleagues discuss the role of transcription factor Bright in both human and mouse B cell development (18), while Serfling and colleagues review the role of NFATc1 transcription factor during hematopoiesis (19).

## Regulation of Class-Switch Recombination and Somatic Hypermutation

Because mature B cells encounter a variety of antigens, they undergo both Class-Switch recombination (CSR) and somatic hypermutation (SHM) to diversify their antibody repertoire by utilizing enzymes such as AID. Given that these processes involve DNA breaks, they must be extremely tightly regulated to maintain genomic integrity (20, 21). Kenter and colleagues (22) and Chaudhuri and colleagues (23) present two articles discussing various factors regulating both SHM and CSR, including three-dimensional genomic topology, chromatin, and transcription. Barbara Birshtein discusses the role of the 3′-enhancer in controlling both SHM and CSR, in particular the epigenetic architecture of the enhancer in these processes (24).

## Transcription Factors Regulating Immune Responses

The ultimate role of immune cells is to mount an effective adaptive or innate response against pathogens (25). Hence, the transcription factors regulating these responses play an extremely important role. The final three articles deal with the transcription factors involved in immune responses and antigen presentation. Corcoran and colleagues present primary data on the function of transcription factor Oct2 and its co-activator Obf1/OCA-B in collaboration between B and T cells during an adaptive immune response (26). Bhatt and Ghosh discuss the role of the critical transcription factor NF-kB in innate immune response and how it controls the process of inflammation, which is crucial in maintaining immune homeostasis (27). Finally, Devaiah and Singer discuss our current understanding of the role of Class II transactivator CIITA (28), which is a master regulator of major histocompatibility complex gene expression necessary for antigen presentation (29).

## Perspective

Mechanisms that regulate communication between enhancers and promoters are complex and involve many transcription factors, accessory molecules and chromatin regulators (30). Given the exquisite timing and precision that are necessary to mount an effective immune response, it is fully anticipated that such complex regulatory mechanisms must be in full display for this to occur. The next few years will undoubtedly uncover more surprises that ultimately will lead to a better understanding of the role of transcription in immune responses.

## Conflict of Interest Statement

The authors declare that the research was conducted in the absence of any commercial or financial relationships that could be construed as a potential conflict of interest.
